# Short-term Effectiveness of Mirikizumab in Crohn’s Disease Patients with Perianal Involvement

**DOI:** 10.31662/jmaj.2026-0215

**Published:** 2026-08-14

**Authors:** Ruby Arai, Kazuyo Okayama, Takanori Nishiguchi, Tetsuro Yamana, Shigeru Kiryu, Masayuki Fukata

**Affiliations:** 1 Center for Inflammatory Bowel Disease, Department of Internal Medicine, Tokyo Yamate Medical Center, Japan Community Healthcare Organization, Tokyo, Japan; 2 Center for Colorectal Surgery, Tokyo Yamate Medical Center, Japan Community Healthcare Organization, Tokyo, Japan; 3 Department of Radiology, International University of Health and Welfare Narita Hospital, Chiba, Japan

**Keywords:** Crohn’s disease, perianal, fistula, mirikizumab, case series

Crohn’s disease (CD) is an idiopathic chronic inflammatory bowel disease characterized by deep transmural ulcerations that can occur anywhere in the gastrointestinal tract ^(1)^. The global prevalence of CD has increased over the last decade. Among Asian patients with CD, including Japanese patients, the frequency of perianal involvement is higher than among patients with CD in Western countries, affecting a substantial proportion of patients ^(1)^. Although medical therapies for intestinal lesions have advanced remarkably ^(1)^, managing perianal disease remains a clinical challenge in patients with CD because it is refractory to most medications.

For a long time, perianal fistulas were primarily managed by definitive surgery or long-term drainage using a seton, a loop-like nonabsorbable thread placed through the fistula tract to prevent recurrent abscesses ^(2)^. However, the introduction of tumor necrosis factor α (TNF-α) inhibitors, a class of biologics, has shifted the paradigm toward medical closure ^(3)^. More recently, biologics targeting interleukin (IL)-23, such as ustekinumab (UST), risankizumab, guselkumab, and mirikizumab (MIRI), have shown efficacy in patients with moderate-to-severe CD who have failed a TNF-α inhibitor ^(4), (5)^. Among these IL-23 inhibitors, MIRI is unique because it is a humanized IgG4 monoclonal antibody that selectively binds the p19 subunit of IL-23, thereby inhibiting the proinflammatory IL-23/T helper 17 (Th17) axis without affecting IL-12–mediated immune responses ^(5)^. However, the specific effect of MIRI on perianal CD has not been investigated. This study retrospectively evaluates the short-term effectiveness and safety of MIRI for perianal CD.

We retrospectively analyzed CD patients with active perianal involvement who received induction therapy with MIRI between May and August 2025. Outcomes were reviewed through November 2025. Data on patient demographics, surgical history, and medications were collected from the electronic medical record. The main local outcomes focused on clinical and radiological improvements in perianal disease, including a reduction in the Perianal Disease Activity Index (PDAI) score, radiological regression assessed using the Van Assche Index ^(6)^, disappearance of drainage, and seton removal. Complete fistula closure was explicitly defined as the absence of active drainage from all external orifices upon gentle digital compression on two consecutive assessments ^(7)^. Concurrently, overall disease activity was evaluated using the Crohn’s Disease Activity Index (CDAI) as a supportive systemic parameter, where a clinical response was defined as a CDAI score reduction ≥70 points or a total score <150. Categorical variables are summarized as frequencies and percentages, and continuous variables are expressed as medians and interquartile ranges (IQRs). Continuous variables and categorical variables were compared using the Wilcoxon signed-rank test and Fisher’s exact test, respectively. Statistical significance was set at p < 0.05.

Sixteen patients were enrolled (Table 1). Prior perianal surgeries included seton placement (n = 9), fistulotomy (n = 2), and incision and drainage for perianal abscesses (n = 5); however, all patients were confirmed by surgeons to have at least one anal fistula. MIRI was initiated at a median of 6 months (IQR 3.5-7.6) after the most recent surgery. Nine patients (56.3%) were biologics (bio)-experienced, having switched from UST (n = 4), adalimumab (n = 3), or vedolizumab (n = 2), while the remaining seven patients (43.8%) were bio-naïve. Five patients received concurrent antibiotics (metronidazole: n = 4; ciprofloxacin: n = 1) at the initiation of MIRI.

**Table 1.** Baseline Characteristics and Treatment Outcomes of Patients (n = 16).

Baseline characteristics	Values
Age (years), median (IQR)	29 (22.0-31.3)
Sex: male, n (%)	15 (93.8)
Disease duration (years), median (IQR)	4.5 (1.6-10.2)
Disease type, n (%)	
Location	
L1	0 (0)
L2	1 (6.3)
L3	15 (93.8)
Behavior	
B1	8 (50)
B2	5 (31.3)
B3	3 (18.8)
CDAI score at initiation of MIRI, median (IQR)	188 (172.0-199.0)
PDAI score at initiation of MIRI, median (IQR)	3 (2.0–5.0)
SES-CD, median (IQR)	10.5 (8.0-18.0)
Van Assche Index score, median (IQR)	12.5 (9.0-13.0)
Previous treatments, n (%)
IFX	0 (0)
ADA	3 (18.8)
UST	4 (25.0)
VDZ	2 (12.5)
Thiopurine	4 (25.0)

ADA: adalimumab; B1: nonstricturing, nonpenetrating; B2: stricturing; B3: penetrating; CDAI: Crohn’s Disease Activity Index; IFX: infliximab; IQR: interquartile range; L1: ileal; L2: colon; L3: ileocolon; MIRI: mirikizumab; PDAI: Perianal Disease Activity Index; SES-CD: Simple Endoscopic Score for Crohn’s Disease; UST: ustekinumab; VDZ: vedolizumab.

Among nine patients with drainage setons, five had their setons removed by the end of the observation period, either spontaneously (n = 1) or by a surgeon due to the absence of discharge (n = 4) (Table 2). The removal or spontaneous drop of the drainage setons indicated a significant reduction or healing of inflammation in the anal fistula. Ultimately, five patients (31.3%) achieved complete fistula closure during a median observation period of 3.9 months (IQR 3.3-3.9) after initiation of MIRI. Reflecting these clinical improvements, the median PDAI score significantly decreased from 3 (IQR 2-5) to 2 (IQR 0.8-3.0) at week 12 of treatment (p < 0.001). Objective assessment based on changes in the Van Assche Index also revealed a significant reduction in the median score from 12.5 (IQR 9-13) to 5 (IQR 3.3-7.0) (p < 0.05) (Figure 1). As a supportive assessment, overall clinical response based on the CDAI was observed in 11 patients (68.8%), with the median CDAI scores decreasing from 188 (IQR 172-199) to 90 (IQR 51.0-156.8) (p < 0.0001). In subgroup analysis, the response rate was higher in bio-naïve patients than in bio-experienced individuals (85.7% vs. 55.6%) (Table 2), although the difference was not statistically significant. Notably, a substantial reduction in the PDAI score was observed only in the bio-naïve group. No adverse events were reported that required MIRI discontinuation.

**Table 2.** Treatment Outcomes after 12 Weeks of MIRI (n = 16).

Outcomes	Values
Perianal disease activity	
Closure of external fistula openings, n (%)	5 (31.3)
Disappearance of discharge, n (%)	5 (31.3)
Seton removal by surgeon, n (%)	4 (25)
Spontaneous seton drop-off, n (%)	1 (6.3)
No change, n (%)	4 (25)
PDAI, median (IQR)	2 (0.8-3.0)
Van Assche Index score, median (IQR)	5 (3.3-7.0)
Overall disease activity	
Clinical response, %	68.8
Clinical remission, %	50
Clinical response (remission) in bio-naive patients, %	85.7 (42.9)
Clinical response (remission) in bio-experienced patients, %	55.6 (55.6)
CDAI, median (IQR)	90 (51.0-156.8)
Adverse events	
Perianal	0
Systemic	0

bio-experienced: previously treated with biologic therapy; bio-naive: previously untreated with biologic therapy; CDAI: Crohn’s Disease Activity Index; IQR: interquartile range; MIRI: mirikizumab; PDAI: Perianal Disease Activity Index.

**Figure 1.**
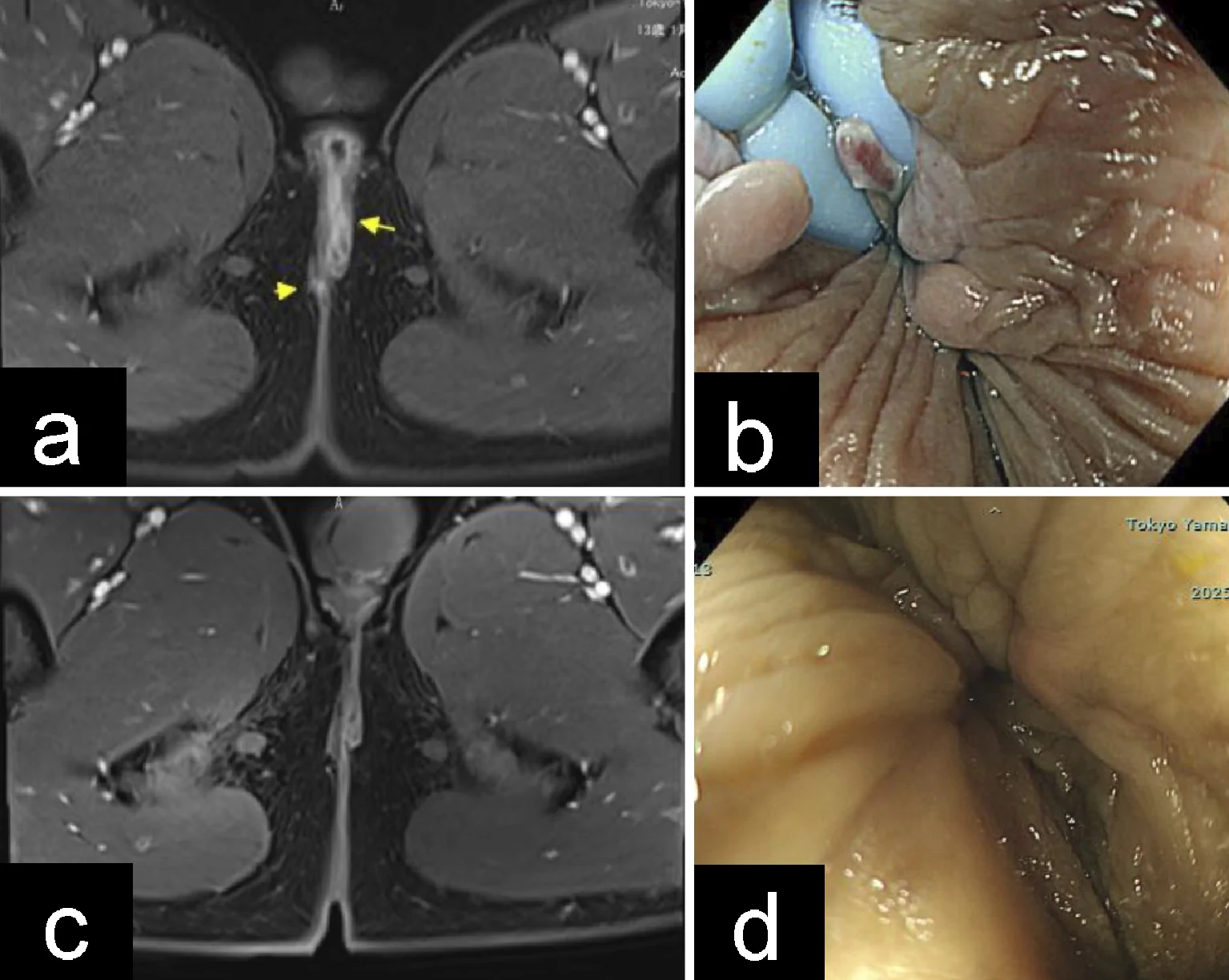
Representative longitudinal course of a patient with perianal Crohn’s disease showing axial fat-suppressed contrast-enhanced T1-weighted MR images and photographs of the perianal lesion. (a) An MR image before treatment. An inflamed anal fistula extending from the anus to the base of the scrotum is delineated as an enhancing lesion (arrows), with abscess formation at the distal tip of the fistula (arrowhead). (b) Two setons were placed, after which MIRI treatment was started. The photograph shows the perianal lesion before MIRI induction, showing an active fistula orifice with two drainage setons in place. Unhealthy granulation tissue is observed around the seton insertion. (c) Setons were removed by the surgeon at week 14 of MIRI treatment. An MR image at week 16 of MIRI treatment demonstrates that the anal fistula has decreased in size, and the abscess is no longer visible. (d) Follow-up photograph of the perianal lesion after MIRI treatment (week 19) showing complete closure of the fistula orifice, with the granulation tissue having disappeared. MIRI: mirikizumab; MR: magnetic resonance.

This case series demonstrated the short-term effectiveness of MIRI, a biological agent that selectively inhibits the p19 subunit of IL-23 and suppresses the IL-23/Th17 inflammatory pathway, for perianal CD. TNF-α inhibitors have been proactively used as the gold standard for perianal CD because of their established efficacy in inducing medical fistula closure. For instance, the ACCENT II trial (A Crohn’s Disease Clinical Trial Evaluating Infliximab in a New Long-Term Treatment Regimen in Patients with Fistulizing Crohn’s Disease) reported a 36% complete fistula closure rate with infliximab maintenance therapy at week 54 ^(8)^. However, secondary loss of response, higher rates of adverse events, and the development of intolerance are ongoing challenges in treatment with TNF-α inhibitors. While UST, an IL-12/23 p40 inhibitor, is frequently used following failure of a TNF-α inhibitor, its efficacy for perianal disease appears to be limited ^(9)^. Meta-analyses have reported a pooled fistula response rate of approximately 40% and a remission (closure) rate of only 17% of UST-treated patients ^(9), (10)^. Despite the shorter follow-up, our 31.3% closure rate compares favorably with reported short-term UST outcomes ^(11)^. Notably, one out of four patients with UST failure in our cohort experienced closure of anal fistula by short-term MIRI treatment.

This clinical outcome is consistent with the recent findings in risankizumab, another IL-23p19 inhibitor, which demonstrated a 28.6% fistula closure rate during the 12-week induction period in a subanalysis of Asian patients ^(12)^. Furthermore, the RESOLVE IG-IBD (Multicenter Real-World Outcomes of Risankizumab in Crohn’s Disease) study showed that risankizumab is safe and effective for refractory perianal CD, with fistula closure rates reaching 30% at week 26 ^(13)^. Therefore, selective IL-23p19 inhibition may be a more effective strategy than inhibiting IL-12/IL-23 in patients with CD with perianal involvement.

Our study cohort included several bio-naïve patients who received MIRI as their initial biologic therapy. While TNF-α inhibitors have commonly been used as first-line biologics for perianal involvement, the favorable safety profile and low immunogenicity of IL-23p19 inhibitors offer an attractive alternative ^(14)^.

This study has several limitations. First, the retrospective, single-center design and small sample size may have introduced selection bias. Second, the lack of a control group makes it difficult to definitively attribute the observed outcomes to MIRI alone. Third, concomitant treatments, including antibiotics and surgical interventions such as the timing of seton placement and removal, may have influenced the outcomes. Finally, the short observation period limits our assessment of the long-term durability of MIRI for fistula closure.

In conclusion, our findings suggest that MIRI may be an additional option for patients with perianal CD. Large-scale prospective studies are warranted to establish the definitive role of IL-23p19 inhibition in achieving long-term remission of CD patients with perianal involvement.

## Article Information

### Acknowledgments

The authors would like to thank all the patients who participated in this study and the staff of Tokyo Yamate Medical Center for their contribution to this work.

### Author Contributions

Concept: Takanori Nishiguchi and Masayuki Fukata; design: Ruby Arai and Masayuki Fukata; data collection and processing: Kazuyo Okayama, Masayuki Fukata, Tetsuro Yamana, and Shigeru Kiryu; analysis and interpretation: Ruby Arai, Masayuki Fukata, and Shigeru Kiryu; literature search: Ruby Arai and Masayuki Fukata; and writing: Ruby Arai, Masayuki Fukata, and Shigeru Kiryu.

### Conflicts of Interest

Masayuki Fukata has received speaker and consultancy fees from Takeda, Mochida, AbbVie, Pfizer, Janssen, BMS, Zeria, Kyorin, Kissei, Nihon Kayaku, and Celltrion. The other authors have no conflicts of interest to declare.

### Ethics Approval

This study protocol was reviewed and approved by the institutional review board of Tokyo Yamate Medical Center (approval number 229).
